# PReS-FINAL-1007: Regulatory T cells functional specialization in JIA

**DOI:** 10.1186/1546-0096-11-S2-P5

**Published:** 2013-12-05

**Authors:** G Mijnheer, B Prakken, FV Wijk

**Affiliations:** 1 Pediatric Immunology, UMC Utrecht, Utrecht, The Netherlands

## Introduction

Regulatory T cells (Treg) are important players in keeping the immune system in balance. In juvenile idiopathic arthritis (JIA), an autoimmune disease characterized by chronic inflammation of the joints, this balance is disturbed. Recently, different functional subsets of regulatory T cells (Treg) have been described in mice and human that mirror the T helper subsets. A lot remains unknown about the function and mechanism of action of Treg (subsets), especially in inflammatory environments.

## Objectives

In our study we aim to investigate the adaptability of regulatory T cells based on phenotype and function. In particular, our focus is on Treg derived from different inflammatory environments.

## Methods

Treg will be isolated from peripheral blood and synovial fluid of JIA patients and peripheral blood of healthy controls and analyzed based on the expression of chemokine receptors CXCR3, CCR6 and CCR4. Currently, autologous suppression assays, allogenic T cell suppression assays and monocyte suppression assays are performed with these Treg subsets derived from different environments.

## Results

Treg subsets that mirror Th subsets can be found and discriminated based on there chemokine receptor profile (i.e. CXCR3, CCR6 and CCR4) in peripheral blood of healthy control and JIA patients, and in the synovial fluid of JIA patients.

## Conclusion

Different subsets of Treg can be identified in the synovial fluid of JIA patients. This allows us to further look in to Treg subset function.

## Disclosure of interest

None declared.

